# Clinical applications of skin traction technique with adjustable tension in treatment of large area skin defects

**DOI:** 10.1186/s12891-023-06628-y

**Published:** 2023-07-06

**Authors:** Md Israil Nadaph, Chong Meng, Xuejian Wu

**Affiliations:** Department of Orthopedics, The First Affiliated Hospital of Zhengzhou University, Zhengzhou, China

**Keywords:** Skin traction, Skin defects, Visual analogue scale, VAS, Skin necrosis, Wound healing

## Abstract

**Objective:**

To explore the clinical applications of the adjustable skin traction technique in the treatment of large area skin defects.

**Research design:**

A prospective study.

**Background:**

The skin is the largest organ of the human body and skin tissue exposed to external environment which makes it vulnerable to damage. There are many reasons for skin defects such as trauma, infection, burns, scars, tumors resection, inflammation, pigmented nevus, etc. Skin traction is the application of pulling force to the trunk or extremities for immobilization, fracture reduction and deformity correction. This technique accurately controls skin expansion which is safe, convenient and accelerates wound healing.

**Methods:**

A prospective study was conducted on 80 patients suffered from large area skin defects in the department of orthopedics, the first affiliated hospital of Zhengzhou University from September 2019 to January 2023. There were 40 patients in the experimental group who underwent skin traction. In contrast, 40 people in the control group underwent skin flaps or skin grafts without skin traction. The inclusion criteria include large area skin defects, normal peripheral skin & blood supply, normal vital organs, no severe coagulation dysfunction etc. Male & female with and without skin traction are 22 & 18 and 25 & 15 respectively. The skin traction device used was a hook and single rod type. The skin defect area was approximately 15 cm × 9-43 cm × 10 cm.

**Results:**

Postoperatively, the experimental group with traction showed 2 cases of skin infection, 1 case of skin necrosis and 3 cases of inflammation recurrence. In contrast, the control group without traction showed 8 cases of skin infection, 6 cases of skin necrosis and 10 cases of inflammation recurrence. Skin infection (*P* = 0.04), skin necrosis (*P* = 0.02) and inflammatory response (*P* = 0.03) represented significant differences between two groups. There was also a significant difference in hospitalization costs (*P* = 0.001).

**Conclusion:**

Skin traction has huge clinical applications including a shorter hospital stay, faster wound healing, lower hospitalization cost, high satisfaction rate, and a fair skin appearance after surgery. It is an effective method of treating skin and musculoskeletal defects.

## Background

Skin defect is also known as cutaneous defect which is any medical condition that affects the integumentary system [1]. A significant aspect of clinical diagnosis of skin defects is gathering pertinent information regarding the presenting skin defect [2]. It’s essential to avoid inappropriate treatment that may delay wound healing, deteriorate skin defects, or harm the patient [3]. The causes of skin defects include infected surgery, trauma, inflammation, burn, skin infection, venous ulcers, pressure ulcers, diabetic foot ulcers and ischemic skin [4]. Untreated skin defect may result in further skin infection or necrosis, which requires treatment for the infection [5]. It is usually associated with significant morbidity, high healthcare costs, loss of productivity and reduced quality of life [6]. Other causes are nephropathy, diabetes, hemophilia or a combination of these diseases. The research report points out that inappropriate treatment of acute trauma is the most common cause of chronic trauma [7]. Research from Chinese studies shows that 67% of skin defects are caused by wounds combined with infection. Diabetes ulcer, venous ulcer and pressure ulcer accounted for 4.9%, 6.5% and 9.2% respectively.

Skin traction is a technique for realigning muscles or dislocated parts of the body by using pulling forces to stretch skin and soft tissues [8]. The use of apparatus to apply skin traction to injured limbs has played a significant role in the treatment of fracture patients since the time of the ancient Greek physician, Hippocrates [9, 10]. The skin traction technique has enormous clinical benefits, including ease of operation, skin stretch resistance, wear resistance, fewer scars, and improved skin shape and colors similar to peripheral skin. This technique accurately controls skin expansion which is safe, convenient and accelerates wound healing. The most common methods to repair skin defects in the extremities and trunk are autogenous skin graft, pedicled skin flap transfer and free skin flap transplantation. The drawbacks of skin flap or skin graft in repairing skin defects include the appearance of the skin flap is bloated, the color is not consistent with the recipient area, the sensory function recovery is poor, not wear resistance, easy to wear and tear in the later stage, damage to the donor area, easy to slip when bearing weight etc.

## Materials and methods

### Design of our adjustable skin traction device

**Fig. 1:**
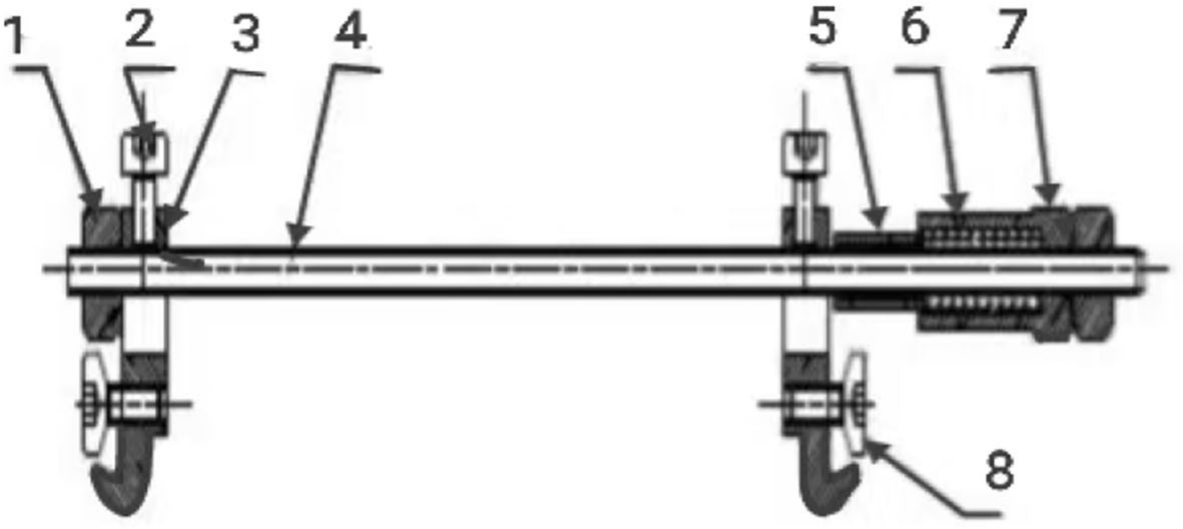
Structure diagram of skin traction device. 1.limit nut, 2-limit screw, 3-fixing hook, 4-threaded rod, 5 -scale tube, 6-spring, 7-adjusting screw sleeve, 8-fixing pin screw

**Fig. 2:**
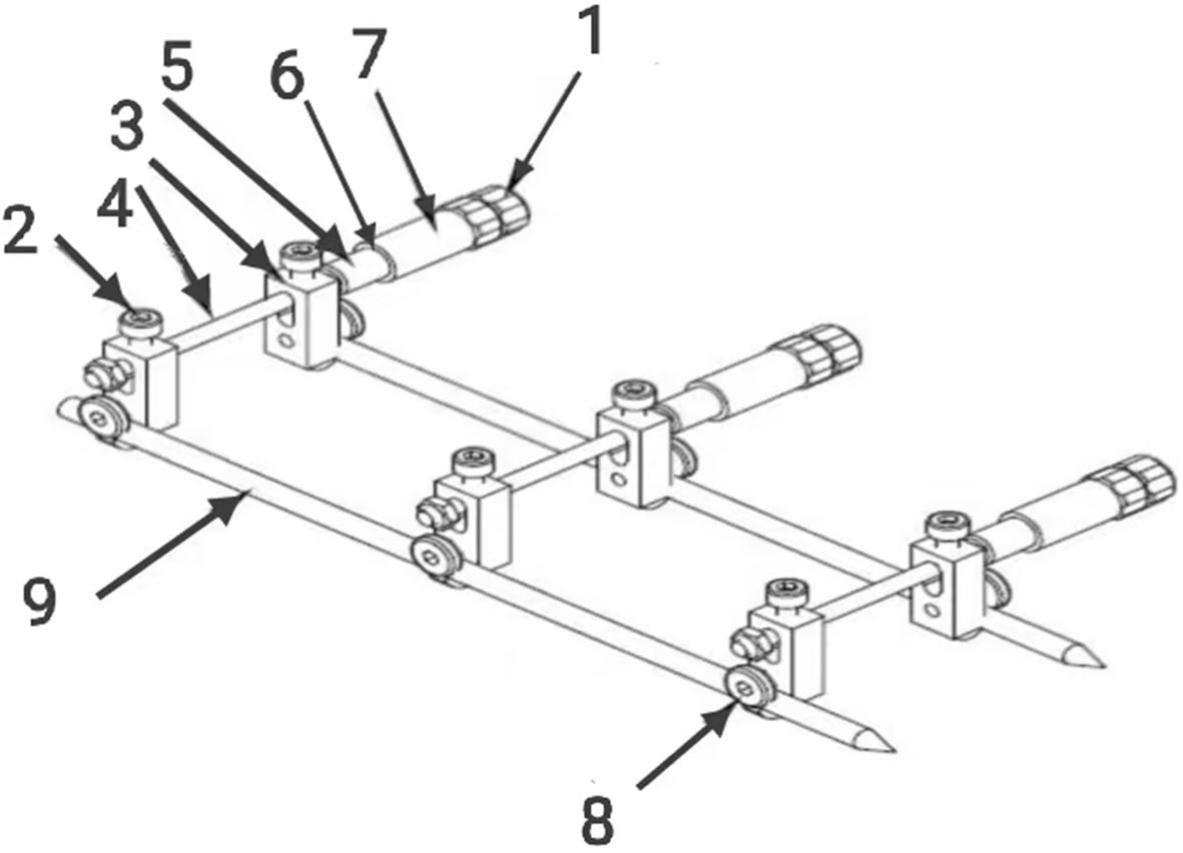
Schematic diagram of skin traction device. 1-limit nut, 2-limit screw, 3-fixing hook, 4-threaded rod, 5-scale tube, 6-spring, 7-adjusting screw sleeve, 8-fixing pin screw, 9-traction pin

**Fig. 3:**
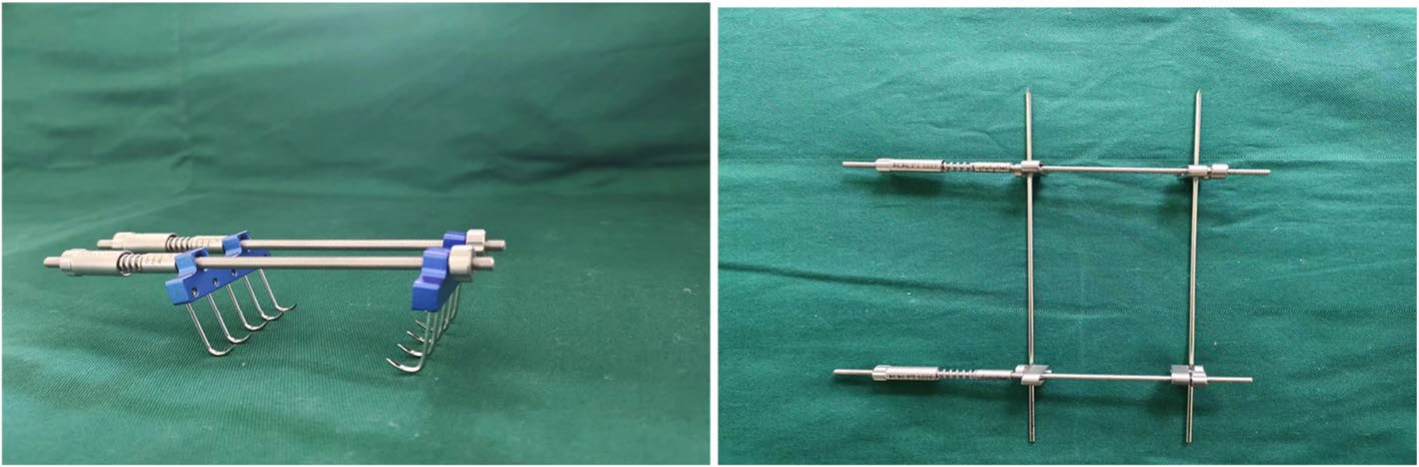
The real design of a skin traction device used in our department of orthopedics

Our adjustable skin traction device is designed and produced by Henan Keke Biotechnology Co. Ltd. that can accurately control skin stretch with adjustable tension which is safe, convenient and has high wound healing speed. According to the needs of patients, this design is relatively flexible and is the most suitable type of traction. It can effectively improve the appearance and function of the recipient area with less damage to the donor area. The main cutaneous nerve & vein are also preserved. The donor area’s venous return is not significantly damaged and the patient recovers quickly after the operation. Therefore, the evaluation of self-made skin traction devices has significant advantages with high treatment satisfaction rates. Figure 1 is a structural diagram while Fig. 2 is a schematic diagram of our skin traction device which we often use in our department. Figure 3 shows the real design of our skin traction device.

Figure 3 illustrates the real design of a skin traction device used in our department of orthopedics. Skin traction device installation requires training and experience because incorrect traction may cause injury [11]. Accurate methods of traction and controlling tension have been developed to provide proper force for maintaining alignment and preventing muscle spasms. Chinese scholars have made enormous progress in researching traction designs to repair skin defects, such as using self-made rubber bands or steel wire combined with Kirschner wire (K-wire). Due to the inability to adjust the tension, these designs are difficult to apply in clinical practice since they cannot be adjusted. By adjusting the tension, the adjustable skin traction device can achieve a wide range of clinical benefits for patients.

### Patient selection

Our current study included 80 patients. They were divided into 40 patients with traction as the experimental group while 40 patients without traction as the control group. Experimental patients have been treated with different forms of single rod type and hook type skin traction device according to different sites of skin defects. The control group is treated with conventional skin grafting or flap transplantation to repair skin defects. To minimize bias and ensure results are not influenced, all patients, researchers, and outcome assessors were blinded to treatment assignment. The demographic information of 80 consecutive patients of both groups suffering from large skin defects caused by trauma, inflammation, tumor resection, pigmented nevus etc. The data were analyzed in the department of orthopedic of the first affiliated hospital of Zhengzhou University from September 2019 to January 2023. Male & female with and without skin traction are 22 & 18 and 25 & 15 respectively. The patients’ age with traction is 25–50 (35 ± 10.5) while without traction is 22–48 (34 ± 8.6) years old. The average skin defect size is 15 cm × 9-43 cm × 10 cm. The medical case history includes 42 cases of skin defects caused by trauma, 8 cases of burn wound, 20 cases of inflammatory skin defects, 6 cases of osteofascial compartment syndrome tension-reducing incision wound, 4 cases of scar wound, including tendon leakage and bone exposure. The patients were followed up for a mean duration of 3–24 months to assess the long-term effectiveness of the skin traction technique. Hook type skin traction was applied in 19 cases, rod type traction in 15 cases, single rod type with external fixation bracket in 4 cases and single rod type & hook type combined traction in 2 cases. The remaining 40 patients in the control group were treated with skin grafting or skin flap transfer. The inclusion and exclusion criteria are as follows:

**Patients’ inclusion criteria**:

- Skin defects caused by trauma, inflammation, tumor resection, pigmented nevus etc.;
- Patients with normal vital organs;
- No severe coagulation dysfunction;
- With normal blood supply and normal skin around skin defects;
- No systemic edema, no severe edema of normal skin around skin defects;
- Free of mental illness.

**Patients’ exclusion criteria**:

- Patients with severe dysfunction of vital organs and cannot tolerate anesthesia or surgery;
- Severe coagulation dysfunction (such as hemophilia);
- Blood circulation disorders around the skin defects;
- Not enough normal skin around skin defects;
- Systemic edema and local skin edema around skin defects;

### Data collection

Patient essential data was collected. A first step in data collection is to collect all the details and information of patients who underwent skin traction as an experimental group. And those who underwent skin grafting & skin flap transfer without traction as a control group. Furthermore, the number of patients, age, gender, visual analogue scale (VAS), hospitalization duration, and healing time for skin defects were determined. The complications of both groups including skin infection, skin necrosis, DVT, pressure ulcers, inflammation, and UTI were noted during treatment.

### Statistical analysis

Data analysis was carried out using SPSS version 28 for Windows (SPSS, Inc, Chicago, IL, USA). Calculation of the healing time of skin defects, the total length of hospitalization, infection and necrosis of skin, the hospitalization cost, and the patient’s satisfaction rate of both groups were studied using SPSS statistical data analysis where values of p < 0.05 represents the significant difference.

### Surgical technique

**Fig. 4:**
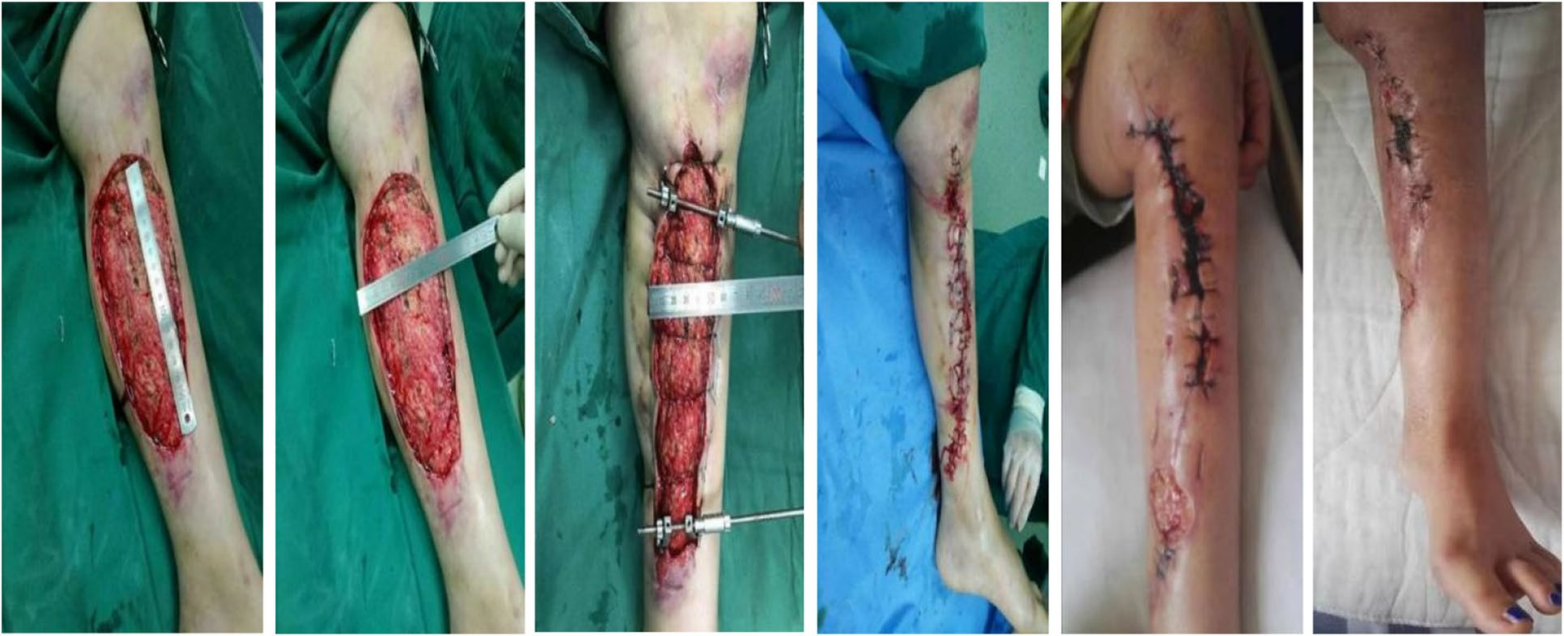
Large area skin defects in left leg due to trauma repaired by skin traction technique

**Fig. 5:**
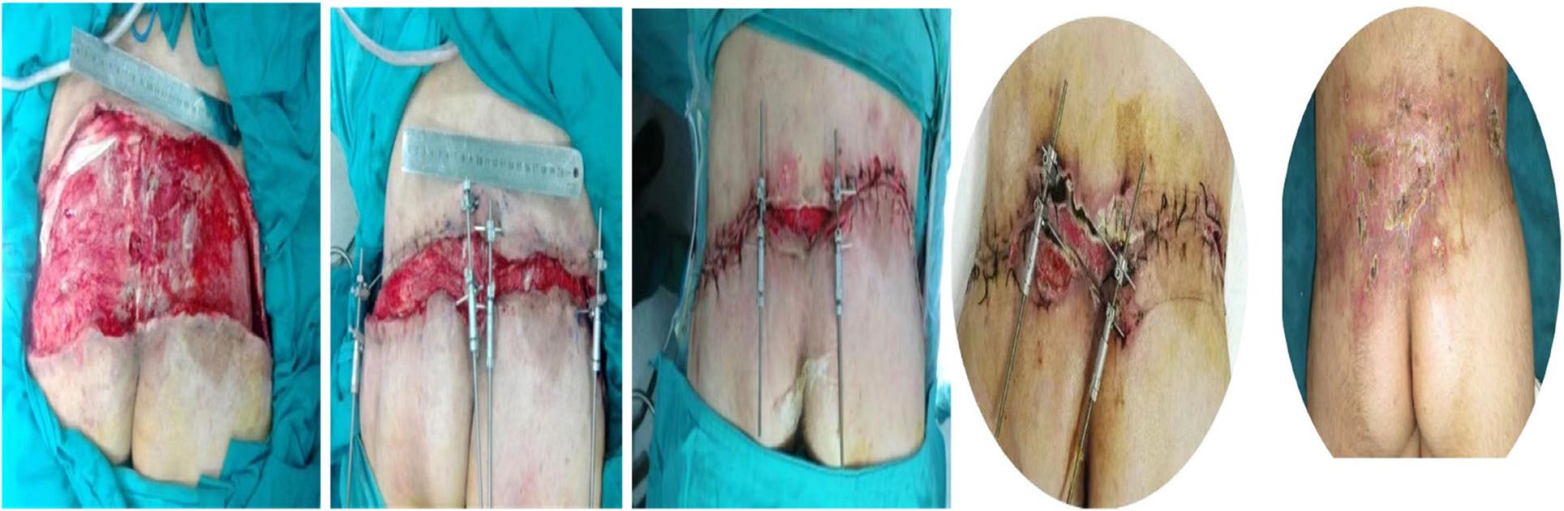
Poor blood circulation site in large area due to accidental trauma repaired by skin traction technique and it’s postoperative view

Skin traction is often used to stabilize skin defects and restore skin tension to the surrounding tissues, muscles, and tendons [2]. In the experimental group, single-rod type skin traction was implanted in parallel on both sides of the wound skin margin from the edge by inserting one traction needle with a diameter of 2-2.5 mm while two row hooks for skin traction were implanted in parallel on both sides of the wound skin margin 1.5 cm from the edge, deep into the superficial fascia layer by an adjusting screw. This adjusting screw can be continuously adjusted 3–4 times per day according to pressure scale changes. During surgery, silk thread can be used to close the skin edges. The control group was not treated with skin traction, and the wound was repaired by traditional methods like skin grafting and a skin flap. After the operation, both groups were routinely treated for inflammation, infection prevention, routine disinfection, and proper sterile dressings. Figure 4 shows a patient underwent skin traction surgery for large area skin defects caused by trauma to the left leg. Figure 5 shows an accident-related poor blood circulation site repaired by traction of the skin.

## Results

2 cases of skin infection, 1 case of skin grafting necrosis, and 3 cases of inflammation recurrence were reported among 40 patients who had undergone skin traction technique in the experimental group. There were 8 cases of skin infection, 6 cases of skin necrosis, and 10 cases of inflammation in the control group of 40 patients who were treated with skin grafting, skin transfer or skin flaps. Patients were followed up for 3–24 months. Patients with skin traction had satisfactory wound healing and the shape & color of treated skin defects were similar to normal skin.

Table 1 shows the general demographic information of patients in both groups. This table illustrates the significant comparison of total duration of hospitalization and skin defects healing time between two groups with *P* < 0.05. So, total duration of hospitalization and skin defects healing time of experimental group are less than control group. The visual Analogue scale (*P* = 0.66) shows no relevant comparison between them.

**Table 1:** Comparison of patients’ demographic information between two groups

Variables	Experimental Group (***n*** = 40)	Control Group (***n*** = 40)	***P***-value
No. of Patients	40	40	0.08
Age in years (Mean ± SD)	35 ± 10.5	34 ± 8.6	0.06
Age Ranging (years)	25-50	22-48	0.06
Gender			
Male	22	25	0.2
Female	18	15	0.4
Mean follow-up (months)	3-24	3-24	0.08
Visual Analogue Scale (VAS)	7±1.0 (3.5-8.0)	5.8±1.0 (1.5-6.0)	0.66
Duration from Injury to admission (hours)	8.5	7.8	0.09
Duration from admission to Surgery (hours)	1.5 ± 0.5	1.4 ± 0.6	0.6
Total Duration of Hospitalization (days)	7 ± 2.5	15 ± 3.5	0.03
Skin defects Healing Time (days)	5 ± 2.0 (2-11)	13 ± 4.0 (8-18)	0.01

By comparing the complications, the no. The total number of patients suffering from skin infection is 2, skin necrosis is 1 and inflammation is 3 in experimental group. However, it is 8, 6 and 10 respectively in control group as shown in Table 2 which represents significant difference among them (*P* < 0.05). While pressure ulcers (*P* = 0.4), DVT (*P* = 0.08) and UTI (*P* = 0.54) demonstrate no statistically significant difference between them. In addition, no other complications were observed in these two groups of patients. Thus, it shows significant differences regarding skin infection, skin necrosis and inflammation while other variables show no significant differences.

**Table 2:** Comparison of complications between patients of both groups

Variables	Experimental Group (***n***=40)	Control Group (***n***=40)	x²	***P***-value
Skin Infection	2	8	0.6	0.04
Skin Necrosis	1	6	0.45	0.02
Pressure Ulcers	10	11	0.2	0.40
Inflammation	3	10	0.6	0.03
DVT	8	9	0.04	0.08
UTI	6	7	0.1	0.54

Table 3 shows significant differences in hospitalization average cost (*P* = 0.001). Patients in the experimental group have a lower hospitalization cost than patients in the control group, which makes them more economically viable and affordable. In addition, it shows a significant difference between the patients’ treatment satisfaction rate in the experimental and control groups (*P* = 0.001), which is 90% in the experimental group and 60% in the control group. Likewise, postoperative surgical site appearance appeared favourable and fair in 38 patients out of 40 in the experimental group. In contrast, there were only 22 patients in the control group. Thus, experimental group patients have significant improvement regarding hospitalization cost, treatment satisfaction rate, and fair postoperative surgical site appearance.

**Table 3:** Hospitalize average cost & treatment satisfaction rate (%) of both groups

Variables	Experimental Group (***n*** = 40)	Control Group (***n*** = 40)	***P***-value
Hospitalization average cost ($)	2000 ± 500	4000 ± 800	0.001
Treatment satisfaction rate (%)	90	60	0.001
Fair post-surgical skin appearance (n)	38	22	0.001

## Discussion

Skin traction is a technique for applying pulling force to the trunk or extremities for immobilization, fracture reduction and deformity correction. The use of apparatus to apply skin traction to injured limbs has played a significant role in the treatment of fracture patients since the Ancient Greek physician, Hippocrates. Skin traction is widely used in plastic and orthopedic surgery. Physical examination of skin defects and their appendages forms the cornerstone of an accurate skin defect diagnosis [12]. Clinical examinations are critical for determining skin defects’ morphology, configuration, and distribution [13]. Skin defects in China are reported at 10–40% [14]. Trauma, hematoma and infection are the most common causes of skin defects [15]. Control group patients with skin defects often require multiple secondary operations after skin flap or skin transfer surgery, which might result in limb slipping. Skin flap surgery has a certain scope. Since the bridge device was developed by Barrier et al. [16] to treat abdominal wounds in 1976, skin traction has been continuously developed in clinical practice. In 1986, Hirshowitz et al. [17] and in 1992, Cohen et al. [18] reported different kinds of skin traction devices to repair skin defects. A skin traction procedure repairs fresh skin defects caused by scarring, tumors, pigmented nevuses etc. on the extremities or trunk. Furthermore, the effects of skin stretching on blood supply were studied by Paolo Erba et al. [19] confirmed that traction induces tissue angiogenesis through mouse model research. The observation of vascular cell morphology showed that the vascular spacing became smaller, the density increased, and the skin after traction showed dense vascular bundles. According to Marquardt, C [20] and other studies, skin elasticity and shear capacity reached the limit when morphological and subcutaneous collagen fibers moved equally. Theoretically, traction tension exceeding physiological endurance will lead to blood supply destruction and hypoxia, and vascular bundle destruction. Skin traction mainly uses the bio-mechanical properties of the skin to stretch the skin which can provide skin tissue with color, texture, thickness and structure similar to the recipient tissue. And most skin expansion preserves sensory nerves, pressure resistance, wear resistance, similar skin color and hair to the surrounding skin. In terms of function, skin traction is significantly superior to skin graft or skin flap transfer. It avoids the donor area damage which is often caused by skin graft or skin flap transfer. In contrast to conventional techniques such as skin grafting and flap transfer, skin traction offers more advantages and has more clinical applications than traditional techniques.

Some small traction devices are mainly applicable to repairing small skin defects, and cannot repair large or complex skin defects. Topclosure® is a plastic stretch band and connecting plate. It can bear limited tension and is only suitable for small wounds or wound tension reduction. Due to the weight bearing function of the lower limbs, the traditional technique causes lower limb dysfunction and related disorders [21–24]. Our skin traction devices can be adjusted accurately, safely, and conveniently even on large area skin defects. This is to ensure traction safety with both single rod and row hook types. It can avoid skin ischemic necrosis due to excessive traction, and also avoid small traction disadvantages. Small traction has several disadvantages, including long traction time and skin infection risk. It has been shown that large skin tractions with adjustable tension can achieve high wound healing speed with decreased risk of complications. These complications including infection, necrosis and inflammation are similar to our study but these complications are often found in procedures without traction. The adjustable traction device controls traction tension. This can directly affect the color, temperature, capillary reaction, and swelling degree at the skin edge. So, it is necessary to monitor the blood supply at the skin edge after operation. And the traction device can be adjusted frequently 3–4 times per day with traction adjustable tension which is generally 2–3 kg.

When used to repair large skin defects on extremities or trunk, the adjustable skin traction technique has many advantages. These include comfort, wear resistance, normal skin color, no damage to the donor area, and relatively simple operation. This technique accurately controls skin tension which is safe, convenient and accelerates wound healing. It repairs medium and large skin defects that cannot be corrected by skin flaps or skin grafting procedures. Skin flap and skin grafting techniques are still useful to repair small skin defects. The disadvantage of skin traction technique is that it is occasionally accompanied with pain during traction, minor traction scars will appear on the skin after operation and inflammation recurrence will occur if the deep inflammatory necrotic tissue is not completely debridement before operation [25]. However, interestingly, the two groups might not need analgesic treatment based on patients’ requests [26]. The limitations of our study include the maximum mechanical force to obtain the highest wound healing rate is not calculated; besides the large area skin defects, the optimal time required to apply the device to other small parts of the body is not observed; the maximum range of traction tension speed to get the highest results has not been studied; it’s not a perfect method with zero damage to components like hair follicles, blood vessels, sweat glands and melanocytes etc. Despite some limitations, skin traction with adjustable tension is one of the most effective ways to treat skin and musculoskeletal disorders.

## Conclusion

Adjustable skin traction is an effective method to treat skin defects by controlling skin expansion which is safe, convenient and accelerates wound healing. It has huge clinical applications such as simple operation, skin stretch resistance, wear resistance, less scars and a better shape & color of skin after recovery. Patients with skin traction require less duration and cost of hospitalization with a high treatment satisfaction rate.

## Data Availability

The datasets generated and/or analyzed during the current study are not publicly available due to prevent copy by others and corresponding author wants it to be privacy but are available from the corresponding author on reasonable request.
